# The power of cognitive rehabilitation in Alzheimer’s disease ‐A Systematic review and meta analysis

**DOI:** 10.1002/alz.095178

**Published:** 2025-01-09

**Authors:** Logien Abuelgasim Ahmad, Mostafa A. Khalifa, Mohammed Alaa Hashad

**Affiliations:** ^1^ Faculty of Medicine, University of Khartoum, Khartoum Sudan; ^2^ Faculty of Medicine, Cairo University, Cairo, Giza Egypt; ^3^ Cairo University, Cairo Egypt

## Abstract

**Background:**

Alzheimer’s disease (AD) is a challenging condition that forbids individuals of their memories and abilities to carry out their daily functioning. In the research for solutions, cognitive rehabilitation (CR) has emerged as a promising intervention to help individuals with Alzheimer’s disease improve their daily activities. Despite its potential benefits, the effectiveness of clinical trials (CT) remains a mystery, inconclusive further investigation. In this study, we aim to unravel the potential of CR in enhancing the lives of individuals with AD, with a focus on evaluating whether CR leads to meaningful enhancements in daily functioning as assessed by validated measures.

**Method:**

Our research is based on the PRISMA guidelines, and to identify relevant studies, a comprehensive search was conducted across multiple databases, including PubMed, Cochrane Library, Scopus, and Web of Science Keywords were used for searching, from inception to 2024. The research was limited to randomized controlled trials examining the effects of cognitive rehabilitation, including a total of 14 clinical trial articles, excluding studies focusing on cognitive stimulation, cognitive training, or cognitive intervention. Non‐English language articles and populations with diagnoses of other conditions are also excluded.

**Result:**

Our systematic reviews yielded a total of 1919 studies. After removing 287 duplicate records, we screened the title and abstract of 1877 papers, excluding 1835. Then, we proceeded to review the full text of 42 articles. 14 of them met the inclusion criteria. A pilot study of four papers is underway, and its results are expected to add to the existing evidence on the effectiveness of cognitive rehabilitation in AD. The meta‐analysis results for other domains of cognitive functions were not statistically complete yet. Further research is warranted to confirm these findings.

**Conclusion:**

Cognitive rehabilitation demonstrated significant positive effects on quality of life and occupational performance in patients with Alzheimer’s disease. Further, research is needed to investigate the efficiency of the intervention in specific domains to enhance understanding of Alzheimer’s disease management.

